# The efficacy and safety of animal-derived nootropics in cognitive disorders: Systematic review and meta-analysis

**DOI:** 10.1016/j.cccb.2021.100012

**Published:** 2021-04-16

**Authors:** Rayan A. Alsulaimani, Terence J. Quinn

**Affiliations:** a Department of Pharmacology, Faculty of Medicine, King Abdul Aziz University, Jeddah 42751, Saudi Arabia; b Institute of Cardiovascular and Medical Sciences, University of Glasgow, Glasgow, UK

**Keywords:** Actovegin, Cerebrolysin, Cortexin, Dementia, Systematic review, Vascular Cognitive Impairment

## Abstract

**Background:**

The animal-derived nootropics, Cerebrolysin, Actovegin and Cortexin, may have potential in treating neurocognitive disorders. Although these drugs have international usage, reports on their efficacy have been conflicting. These agents have been suggested for all dementia types, but may have particular utility in vascular cognitive impairment (VCI). We used systematic review and meta-analyses to summarize evidence of efficacy in all cause dementia, with a subgroup analysis restricted to VCI.

**Methods:**

We searched multidisciplinary, electronic databases from inception to August 2020. We assessed risk of bias using the Cochrane tool and framed results using GRADE criteria. We used random effects models to create summary estimates. Our primary outcome was change in cognition based on any quantitative cognitive assessment scale using standardized mean difference (SMD). We assessed various secondary efficacy outcomes and a safety outcome of serious adverse events. We performed subgroup analyses limited to VCI.

**Results:**

Summary estimates suggested Cerebrolysin was potentially beneficial in improving cognition (8 trials,793 participants, SMD:-0.16, 95%CI:-0.30 to -0.03) and global function (4 trials,479 participants, OR:2.64, 95%CI:1.17 to 5.98) in the short term. There was no difference in incidence of serious adverse events (6 trials,1014 participants, RR:0.96, 95%CI:0.78 to 1.18). In our VCI subgroup, results were similar to the main analysis, with a beneficial effect on cognition (SMD:-0.22, 95%CI:-0.42 to -0.03) and clinical global impression (OR 2.99, 95%CI:1.02 to 8.73). The limited number of eligible studies for Actovegin (*n* = 2 trials,563 participants) and Cortexin, (*n* = 1 trial,80 participants) precluded meta-analyses but data suggested potential efficacy and no safety concerns. Across all included studies, risk of bias was moderate to high, there was imprecision, and certainty of evidence was considered low to very low.

**Conclusion:**

Although published data suggest potential benefits and relative safety of animal derived nootropics, the supporting evidence is weak. The size of the effects demonstrated were modest and probably less than would be considered clinically relevant.

## Highlights

• Animal-derived nootropics may have potential in treating cognitive disorders, especially vascular cognitive impairment.
• Despite widespread use, there are few randomized controlled trials on animal derived nootropics for cognitive disorders.
• Our review suggested modest beneficial effects of these nootropics, but the strength of supporting evidence was limited.
• The clinical significance of the reviewed nootropics in treating vascular cognitive impairment remains unclear.
• The findings of this review indicate promising evidence to justify further large-scale randomized controlled trials.

## 1 Introduction

Dementia and other neurocognitive disorders represent a growing global health challenge [1]. The economic implications are substantial with global cost of dementia predicted to exceed US $1 trillion [2]. In this context, treatments that can prevent, delay or lessen the effects of dementia are urgently needed. Unfortunately, approved therapies, such as Acetylcholinesterase inhibitors and the NMDA receptor antagonist Memantine, are few and only provide symptomatic relief for some patients, with no apparent survival benefits. Furthermore, these drugs are predominantly utilized in treating Alzheimer's disease (AD), whereas available options to manage vascular cognitive impairment (VCI) are even more limited [3]. With so few treatment options, especially for VCI, there is a pressing need to find effective interventions.

Nootropics may represent a promising approach for cognitive disorders [4]. Broadly, nootropics refer to a class of synthetic or natural cognitive-enhancing compounds used to boost mental function [5]. The drugs Cerebrolysin, Cortexin and Actovegin are the most commonly used animal-derived nootropics. Cerebrolysin and Cortexin are neuropeptide preparations consisting of biologically active peptides isolated from porcine brains [6,7]. Actovegin is a highly purified extract of calf's blood comprising a mixture of over 200 biologically active components [8]. The exact mechanism of action of these agents is uncertain and pleiotropic effects including neuroprotection, regulation of neuronal metabolism, and neurotrophic activity have been postulated [7,9,10]. Direct and indirect effects of the nootropics on the cerebrovasculature have been described and some studies have suggested beneficial effects in stroke recovery. These observations suggest that nootropics may have particular benefits in those cognitive syndromes with a vascular basis.

Currently, Cerebrolysin is used to treat traumatic brain injury, stroke, and dementia in over 45 countries including Russia and China [11,12]. Actovegin has been utilized for similar neurological conditions for nearly 50 years [8,9] and is licensed for use in Russia, Eastern European and Asian countries [8]. Cortexin is predominantly prescribed in Russia, where it used to treat a range of acute and chronic neurological disorders including stroke and cognitive impairment [10,13].

To date, no available drugs are clinically proven to prevent the progression of cognitive decline nor restore cognitive function in people with a post-stroke dementia [8]. Although the exact nature of nootropic effects remains a source of debate, a potential effect on vascular function has been postulated and indeed many of these agents are used in cerebrovascular conditions such as stroke. Given the limited therapeutic options to manage VCI, nootropics may represent a promising approach to treating such conditions [7]. While numerous clinical trials have suggested favorable benefit-risk ratios of the animal derived nootropics, available evidence is accompanied by several limitations [13,8,14–16]. Despite the extensive use of animal-derived nootropics in international healthcare, current evidence regarding their efficacy and safety is conflicting. We aimed to synthesize the published evidence relating to efficacy and safety of these three pre-specified animal-derived nootropics, using contemporary approaches for risk of bias, meta-analyses and framing the quality of results.

## 2 Methods

Our systematic review adheres to the Preferred Reporting in Systematic Review and Meta-Analyses (PRISMA) 2009 guidance [17,18]. All aspects of search strategy, title selection, data extraction, and risk of bias assessment (RoB) were independently performed by the two review authors with disagreement resolved through discussion. Our search strategy followed best practice in systematic review and used validated search syntax where available. We performed three reviews, one for each agent. Our review questions used the PICOS framework [19]. Our primary review question was: What is the effect of Cerebrolysin, Actovegin and Cortexin compared to placebo and/or standard care on cognitive outcomes and we pre-specified a subgroup analysis restricted to VCI.

### 2.1 Eligibility criteria

We included randomized controlled trials (RCTs) with full text publications. We set no restrictions on year of publication or language. We included participants with cognitive impairment of any severity and clinical dementia diagnosis using validated criteria. We set no restrictions on dosage regimen, route of administration, or duration of therapy for Cerebrolysin, Actovegin and Cortexin.

### 2.2 Outcome measures

Our primary outcome measure was change in cognitive performance and could be measured by any multidomain cognitive assessment tools.

We pre-specified a series of secondary efficacy measures:

• Clinical global function.
• Behavioral and psychological symptoms of dementia (BPSD).
• Activities of Daily Living (ADL).
• Quality of life (QoL).
• Caregiver burden.

For safety measures we included reports of serious adverse events (SAEs) using standard definitions.

### 2.3 Search strategy

We searched six multidisciplinary, international databases from inception to August 2020: Medline (Ovid), Embase (Ovid), APA PsycInfo (EBSCOhost), Cumulative Index to Nursing and Allied Health Literature (CINAHL) (EBSCOhost), Literatura Latino Americana em Ciências da Saúde (LILACS) (Bireme), and China National Knowledge Infrastructure (CNKI) (CNKI.net).

We also searched ClinicalTrials.gov for registered and ongoing RCTs. The full syntax of the Medline search is available as supplementary materials (Tables I–III).

We imported titles into Mendeley Desktop software (Elsevier, version 1.19.4) [20]. We screened and reviewed titles and abstracts discarding irrelevant titles, and then screened remaining full texts. We created PRISMA flow diagrams for each nootropic of interest [18].

From eligible studies, we recorded details on sampling frame, number included, outcome measures, treatment regimen, comparator, and trial duration.

### 2.4 Risk of bias and quality of evidence

We assessed each included study for RoB using the Revised Cochrane tool for RCTs (RoB-2) [21]. This tool evaluates bias arising from: the randomization process; deviations from intended interventions; missing/incomplete outcome data; measurement of the outcome; selective reporting. We categorized global RoB as ‘low’ if all individual domains were rated low. We categorized as ‘some concern’ if at least one domain had concerns but no domains were rated high risk. We categorized as ‘high risk’ if high RoB was recorded for at least one domain or if multiple domains had some concern. We generated RoB summary figures using the robvis data visualization tool [22].

We assessed for publication bias through symmetry of funnel plots (RevMan 5.4 software) [23].

We assessed heterogeneity of effect using the *I*^2^ statistic, where a value greater than 50 suggested substantial heterogeneity. We used subgroup analyses to explore sources of heterogeneity where applicable. We pre-specified a subgroup of interest around studies with a VCI focus and, where data allowed, we replicate our analyses limited to those papers with a VCI population.

We described certainty of evidence based on five considerations of the Grading of Recommendations, Assessment, Development and Evaluations (GRADE) approach: study limitations, inconsistency of effect, imprecision, indirectness, and publication bias [24]. We created summary of findings tables using GRADEpro GDT software (version 3.6, Evidence Prime) [25].

### 2.5 Measures of treatment effect

Where data allowed we performed meta-analyses using Cochrane Review Manager (RevMan 5.4) software [23]. For continuous outcomes, we recorded mean, standard deviation (SD), and sample size (*n*) of intervention and control groups. For articles reporting standard error (SE) or 95% confidence intervals (95% CIs), rather than the SD, the generic inverse variance method was used to derive SD [23]. We described results of continuous outcomes as mean differences (MD) with corresponding 95% CIs. For those outcomes where the construct was assessed using varying measures, we used standardized mean difference (SMD). Where there were differences in direction of the scale, values with positive scores indicating improvement, were multiplied by -1 to allow consistency of direction of effect.

For dichotomous outcomes, we recorded number of events of interest and the total number of participants, in both intervention and control groups. Results of dichotomous outcomes were presented as odds ratios (ORs) and risk ratios (RRs) with corresponding 95% CIs.

## 3 Results

### 3.1 Search results

**Fig. 1:**
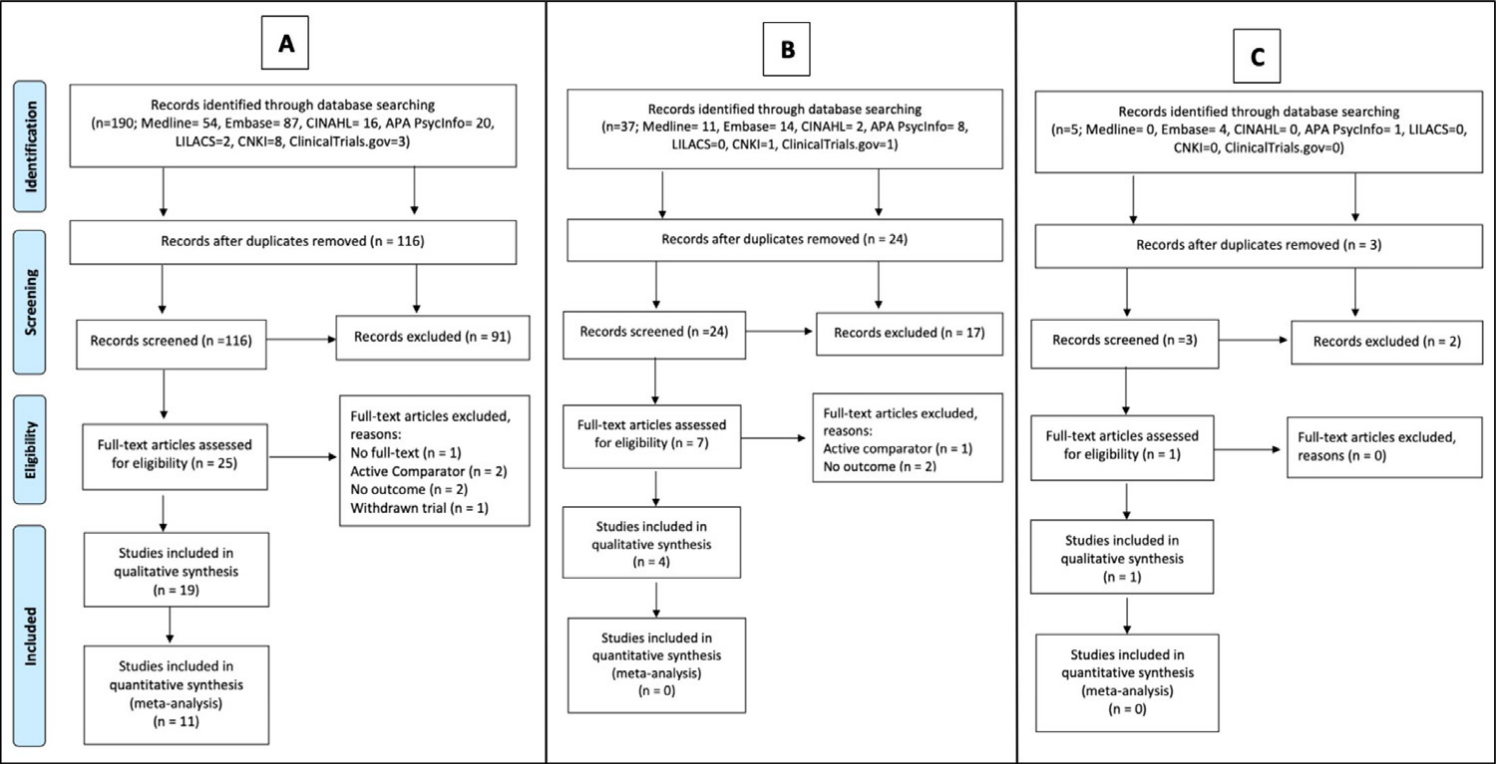
Prisma flow diagrams describing details of the Cerebrolysin (A), Actovegin (B), and Cortexin (C) studies selection process.

From 187 titles, we included 19 papers (1602 participants) pertaining to Cerebrolysin (Fig. 1A). From 36 titles we included four papers pertaining to Actovegin (563 participants) (Fig. 1B). From five titles, we included one paper pertaining to Cortexin (80 participants) (Fig. 1C).

Characteristics of included Cerebrolysin, Actovegin and Cortexin studies are summarized in Table 1.

**Table 1:** Summary of study characteristics of all included Cerebrolysin, Actovegin and Cortexin trials.

Author (Year)	Country	Sample size	Mean age, year^1^	Patient population	Primary variable	Secondary variable	Treatment regimen^2^	Comparator	Trial duration
**Cerebrolysin trials**									
Guekht et al. (2011)	Russia	242	67.3 ± 8.0	Age: 50–85, mild to moderate VaD	ADAS-Cog+, CIBIC+	MMSE, ADCS-ADL, Clock-Drawing Test, Trail-Making Test.	20 ml/day, 5 days/week for 4 weeks, repeated after 2 months of treatment-free interval + daily 100 mg ASA	Placebo (saline)	24 weeks
Muresanu et al. (2008)	Not indicated	41	70.7 ± 10.3	Age: 51–88, mild to moderate VaD	ADAS-Cog+	N/A	10 or 30 ml/day, 5 days/week	Placebo (saline)	4 weeks
Xiao et al. (1999)	China	147	69.7 ± 6.8	Age: 55–85, mild to moderate VaD	MMSE, CGI	SCAG, NAI, Katz-ADL, ZVT, HAMD	30 ml/day, 5 days/week	Placebo (saline)	4 weeks
Alvarez et al. (2011)	Spain	130	75.6 ± 7.6	Age: ≥ 50, mild to moderate AD	ADAS-Cog+, CIBIC+	ADCS- ADL, NPI	10 ml/day, 5 days/week for 4 weeks, repeated after 2 months of treatment-free interval.	Donepezil	28 weeks
Alvarez et al. (2006)	Spain	279	73.6 ± 8.3	Age: ≥ 50, mild to moderate AD	ADAS-Cog+, CIBIC+	MMSE, NPI, DAD	10, 30 or 60 ml/day, 5 days/week for 4 weeks, then 2 days/week for 8 weeks	Placebo (saline)	24 weeks
Muresanu et al. (2002)	Not indicated	60	Not indicated	Age: 50–80, mild to moderate AD	ADAS-Cog+,	CIBIC+, DAD	30 ml/day, 5 days/week for 6 weeks	Placebo (saline)	18 weeks
Panisset et al. (2002)	Canada	192	74.2 ± 6.3	Age: ≥ 60, mild to moderate AD	ADAS-Cog, CIBIC+	MMSE, DAD, Behave-AD, IADL, CDR	30 ml/day, 5 days/week	Placebo (saline)	24 weeks
Ruether et al. (2001)	Germany, Austria	149	73.0 ± 7.7	Age: 50–85, mild to moderate AD	ADAS-Cog, CGI	SKT, MADR-S, NAI	30 ml/day, 5 days/week for 4 weeks, repeated after 2 months of treatment-free interval	Placebo (saline)	28 weeks
Shifu et al. (2000)	China	157	70.3 ±7.7	Age: 55–85, mild to moderate AD	MMSE, CGI	SCAG, NAI, Katz-ADL, ZVT, HAMD	30 ml/day, 5 days/week	Placebo (saline)	4 weeks
Bae et al. (2000)	South Korea	53	71.6 ± 9.6	Age: ≥ 50, mild to moderate AD	ADAS-Cog, CGI	MMSE, GDS, IADL, Katz-ADL	30 ml/day, 5 days/week	Placebo (saline)	4 weeks
Rüther et al. (1994)	Germany	120	71.5 ± 8.3	Age: 55–85, mild to moderate AD	SCAG, CGI, ZVT	NAI	30 ml/day, 5 days/week	Placebo (saline)	4 weeks
Chen et al. (2013)	China	32	44.8 ±16.36	Age: 30–75, mild TBI	MMSE, CASI	N/A	30 ml/day, 5 days/week for one week	Placebo (saline)	12 weeks
**Actovegin trials**									
Guekht et al. (2017)	Russia, Belarus, Kazakhstan	503	69.9 ± 7.0	Age: ≥60, ≤7 days after mild to moderate PSCI	ADAS-Cog+	MoCA, BDI-II, EQ-5D	250 ml/day for ≤20 intravenous infusions followed by 1200 mg/day orally administered for 6 months	Placebo	12 months
Kanowski et al. (1995)	Not indicated	60	80.4 ± 6.5	Age: ≥60, OBS	CGI, SCAG	SKT	250 ml/day intravenous infusions for 4 weeks	Placebo (saline)	1 month
**Cortexin trials**									
Evzel'man and Aleksandrova (2015)	Russia	80	59.3 ± 6.6	Age: 30–73, mild to moderate PSCI	MMSE, 10-word memory test, MoCA	N/A	10 mg/day, 10 days, then regimen was repeated 3, 6 and 9 months following the 1st dose + basic therapy (intramuscular)	Basic therapy	12 months

1 Values are mean ± SD.

2 Cerebrolysin and Actovegin administered intravenously, and Cortexin administered intramuscularly. ***Abbreviations****:* AD: alzheimer's disease; ADAS-Cog: alzheimer's disease assessment scale-cognitive subscale; ADAS-Cog+: alzheimer's disease assessment scale-cognitive subscale plus; ADCS-ADL: alzheimer's disease cooperative study– activities of daily living; BDI-II: beck depression inventory-II;behave-AD: behavioral symptoms in alzheimer's disease; CASI: cognitive abilities screening instrument; CDR: clinical dementia rating scale; CGI: clinician global impression; CIBIC+: clinician interview-based impression of change; DAD: disability assessment in dementia; EQ-5D: euroqol questionnaire; GDS: global deterioration scale; HAMD: hamilton depression scale; IADL: instrumental activities of daily living; Katz-ADL: katz index of independence in activities of daily living; MADR-S: montgomery asberg depression rating scale; MMSE: mini-mental state examination; MoCA: montreal cognitive assessment scale; NPI: neuropsychiatric inventory; NAI: nuremberg activities inventory; PSCI: post-stroke cognitive impairment; SCAG: sandoz clinical assessment-geriatric scale; SKT: syndrome-short-test; TBI: traumatic brain injury; VaD: vascular dementia; ZVT: zahlen-verbindungs-test (Trail-making test).

### 3.2 Risk of bias assessment

#### 3.2.1 Cerebrolysin

**Fig. 2:**
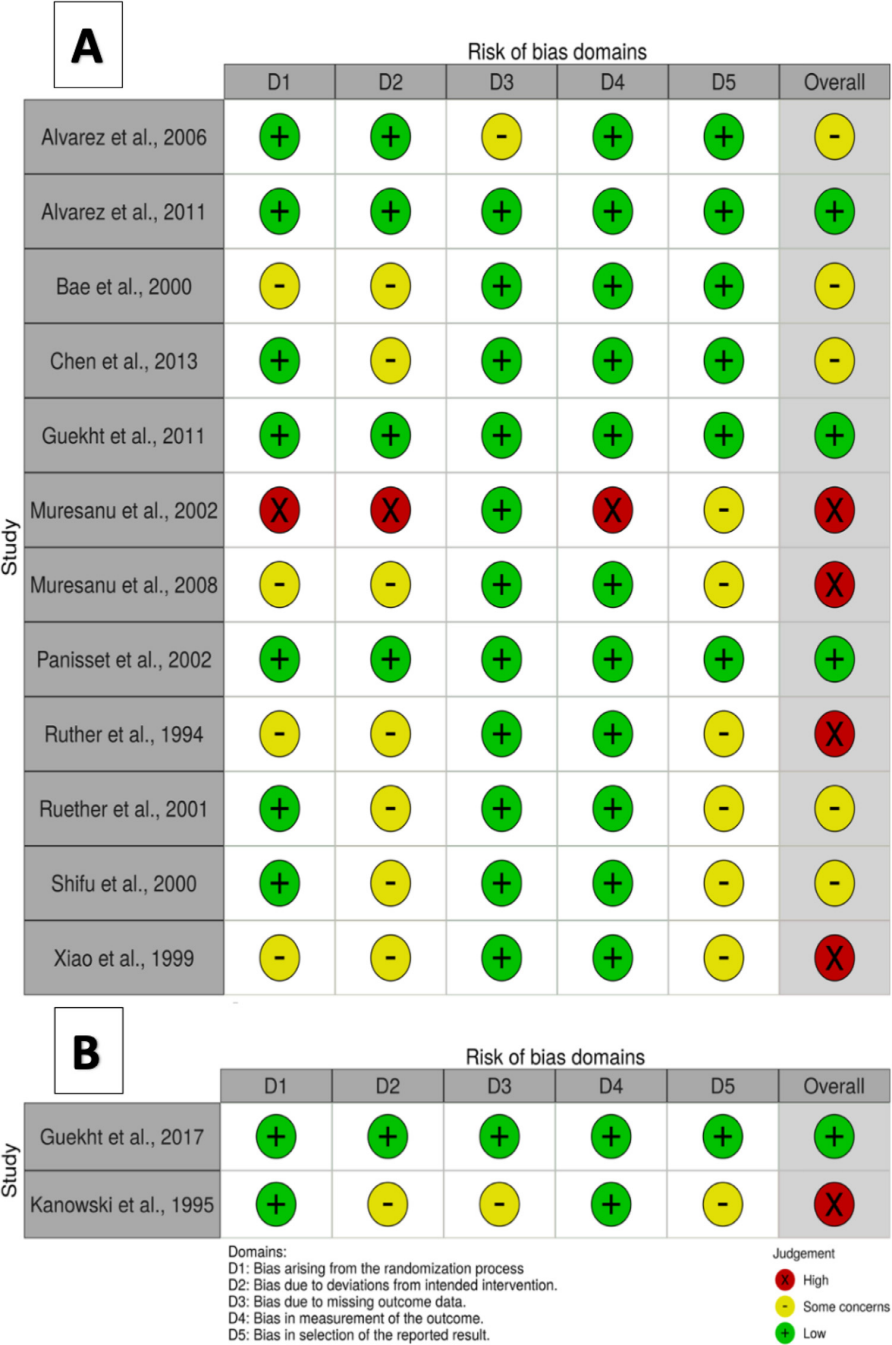
Summary of risk of bias ratings of each bias domain for each included Cerebrolysin (A), and Actovegin (B) trials.

For four studies, the randomization domain was rated as ‘some concerns’ as the published report provided limited details on randomization methods [15,16,26,27]. One study was judged high risk of selection bias as it was an open label study and baseline characteristics were not provided [28]. Seven studies were rated as ‘some concerns’ in the deviations from intended interventions domain as they did not account for important baseline differences in the analyses [15,16,26,27,29–31]. One study was judged high risk because it was not blinded and did not account for baseline differences [28]. In one study, attrition bias was rated as ‘some concerns’, as loss to follow-up was 42 out of 279 (15%) with inconsistencies in the reasons for discontinuation and proportions of missing data (10% (Cerebrolysin) vs. 19% (Control)) [32]. One study was judged high risk for outcome assessment as assessors were not blinded [28]. Six studies were graded as ‘some concerns’ for selective reporting as there was no available protocol and details in the published report were inadequate [15,16,27,28,30,31]. RoB ratings are illustrated at individual study level (Fig. 2A) and in aggregate (Fig. I; available as supplementary materials).

#### 3.2.2 Actovegin

One study was graded ‘some concerns’ for deviation from intended interventions domain as the analysis did not account for important baseline differences [33]. One study was graded ‘some concerns’ for the attrition domain, as the number lost to follow up was 8 out of 60 (13%) and there were differences in the reasons for discontinuation and proportion of missing data (5% (Actovegin) vs. 30% (Control)) [33]. One study was graded ‘some concerns’ around selective reporting as there was no protocol and details in the published report were inadequate [33].

RoB ratings are illustrated at individual study level (Fig. 2B) and in aggregate (Fig. II; available as supplementary materials).

#### 3.2.3 Cortexin

For the one eligible study, the randomization process was graded ‘some concerns’, as details pertaining to random sequence generation, allocation concealment and baseline characteristics were not described. The included study was judged to be at high RoB in the deviation from intended intervention domain, as information about participants and trial personnel blinding, and the analyses utilized to estimate the effect of the intervention were not provided. The included trial was at high risk of attrition bias because the extent of missing data was not reported for one outcome and partially reported for the other outcome. Details pertaining to the blinding of outcome assessors were not provided, and therefore, it was judged to be at high RoB in this domain. The study was graded ‘some concerns’ for selective reporting as there was no protocol and details in the published report were inadequate.

RoB ratings are illustrated at individual study level (figure III; available as supplementary materials).

### 3.3 Effects of interventions

#### 3.3.1 Cerebrolysin

*Cognitive function:* Across the included studies, cognitive performance was assessed using different instruments. The Alzheimer's Disease Assessment Scales (ADAS-Cog/ADAS-Cog+) were used in eight studies [14,15,26,28,30,32,34,35]; and Folstein's Mini-Mental State Examination (MMSE) in seven studies [14,16,26,29,31,32,35]. Two studies were not included in the meta-analysis as one used an active control [34]; and the other study evaluated a different dose of Cerebrolysin (20 ml) to all the other trials [14]. The remaining RCTs all evaluated 30 ml/day of Intravenous Cerebrolysin compared to placebo.

**Fig. 3:**
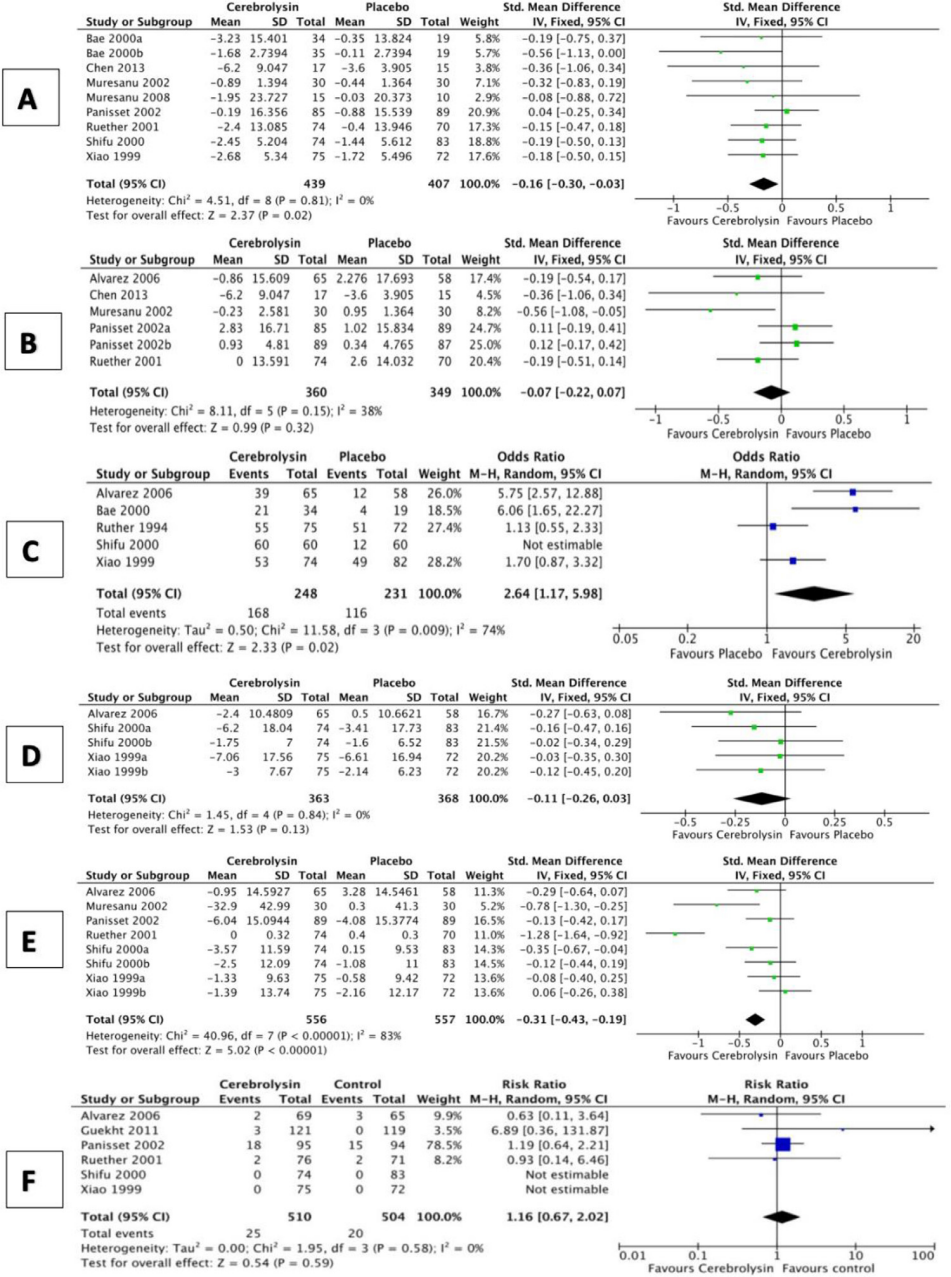
Forest plots comparing the change of various outcomes from baseline in patients receiving Cerebrolysin (30 ml/day) vs. placebo. Cognitive function short term effects (A); Cognitive function medium term effects (B); clinical global impression(C); BPSD (D); ADL (E); the incidence of serious adverse events (F).

We quantified two summary measures of effect to account for the differing timeframes at which outcome data were collected. Short term effects (less than four weeks from initiation of Cerebrolysin) were assessed in 793 participants from eight studies, of which 404 received Cerebrolysin. There was a SMD of -0.16-point (95%CI:-0.30 to -0.03; *P* = 0.02; Fig. 3A) in favor of Cerebrolysin. This improvement was consistent across the included studies (*I*^2^=0). There was substantial funnel plot asymmetry suggesting publication bias (Fig. IV(A); available as supplementary materials). The certainty of evidence was judged to be very low due to serious RoB, imprecision, and suspected publication bias (Table 2). Medium term effects (3 to 7 months from initiation of Cerebrolysin) were assessed in 537 participants from five studies, of which 275 received Cerebrolysin. There was no apparent effect, a SMD of -0.07-point (95%CI:-0.22 to 0.07; *P* = 0.32; Fig. 3B). Moderate statistical heterogeneity was observed (*I*^2^ = 38%). There was substantial funnel plot asymmetry suggesting publication bias (Fig. IV(B); available as supplementary materials). The certainty of evidence was judged to be very low due to serious RoB, inconsistency, imprecision, and suspected publication bias (Table 2).

**Table 2:** Summary of Findings.

Cerebrolysin compared to placebo or routine care for neurocognitive disorders											
Certainty assessment							Summary of findings				
Participants (studies)Follow up	Risk of bias	Inconsistency	Indirectness	Imprecision	Publication bias	Overall certainty of evidence	Study event rates (%)		Relative effect(95% CI)	Anticipated absolute effects	
							With placebo or routine care	With Cerebrolysin		Risk with placebo or routine care	Risk difference with Cerebrolysin
**Cognitive function (follow up: range 3 weeks to 4 weeks; assessed with: ADAS-cog, ADAS-cog+ or MMSE)**											
793(8 RCTs)	serious a	not serious	not serious	serious b	publication bias strongly suspected c	⨁◯◯◯VERY LOW	388	405	-	-	SMD **0.16 SD lower**(0.3 lower to 0.03 higher)
**Cognitive function (follow up: range 3 months to 7 months; assessed with: ADAS-cog, ADAS-cog+ or MMSE)**											
537(5 RCTs)	serious d	serious e	not serious	very serious f	publication bias strongly suspected c	⨁◯◯◯VERY LOW	262	275	-	-	SMD **0.07 SD lower**(0.22 lower to 0.07 higher)
**Global function (follow up: range 4 weeks to 24 weeks; assessed with: CGI or CIBIC+)**											
479(5 RCTs)	very serious g	very serious h	not serious	serious b	none	⨁◯◯◯VERY LOW	116/231 (50.2%)	168/248 (67.7%)	**OR 2.64**(1.17 to 5.98)	502 per 1,000	**225 more per 1,000**(from 39 more to 356 more)
**Behavioral and psychological symptoms (follow up: range 4 weeks to 24 weeks; assessed with: NPI, SCAG or HAMD)**											
427(3 RCTs)	very serious i	not serious	not serious	serious f	none	⨁◯◯◯VERY LOW	213	214	-	-	SMD **0.11 SD lower**(0.26 lower to 0.03 higher)
**Activities of daily living (follow up: range 4 weeks to 28 weeks; assessed with: DAD, NAI or ADL-Katz)**											
809(6 RCTs)	serious j	very serious k	not serious	serious b	publication bias strongly suspected c	⨁◯◯◯VERY LOW	402	407	-	-	SMD **0.31 SD lower**(0.43 lower to 0.19 lower)
**Serious adverse events (follow up: range 4 weeks to 28 weeks)**											
1014(6 RCTs)	serious l	not serious	not serious	serious b	none	⨁⨁◯◯LOW	20/504 (4.0%)	25/510 (4.9%)	**RR 1.16**(0.67 to 2.02)	40 per 1,000	**6 more per 1,000**(from 13 fewer to 40 more)

**CI:** Confidence interval; **SMD:** Standardised mean difference; **OR:** Odds ratio; **RR:** Risk ratio

Explanations

a Downgraded one level for risk of bias: four trials raised some concerns, three trials were at high risk of bias, and one trial had low risk of bias. Most of the 'Risk of bias' judgements were at moderate to high risk of bias.

b Downgraded one level for imprecision: the confidence intervals in most of included trials are wide.

c Downgraded for publication bias due to asymmetry in the funnel plot.

d Downgraded one level for risk of bias: three trials raised some concerns, one trial was at high risk of bias, and one trial had low risk of bias. Most of the 'Risk of bias' judgements were at moderate to high risk of bias.

e Downgraded one level for inconsistency: there was moderate statistical heterogeneity (*I²* = 38%), inconsistency in point estimates and time to follow-up.

f Downgraded two levels for imprecision: the confidence intervals in most of included trials are wide and the result was not statistically significant

g Downgraded two levels for risk of bias: three trials raised some concerns and two trials were at high risk of bias. All of the 'Risk of bias' judgements were at moderate to high risk of bias.

h Downgraded two levels for inconsistency: there was significant statistical heterogeneity (*I²* = 74%), inconsistency in point estimates, and time to follow-up.

i Downgraded two levels for risk of bias: two trials raised some concerns and one trial was at high risk of bias. All of the 'Risk of bias' judgements were at moderate to high risk of bias.

j Downgraded one level for risk of bias: three trials raised some concerns, two trials were at high risk of bias, and one trial had low risk of bias. Most of the 'Risk of bias' judgements were at moderate to high risk of bias.

k Downgraded two levels for inconsistency: there was significant statistical heterogeneity (*I²* = 83%), inconsistency in point estimates, and time to follow-up.

l Downgraded one level for risk of bias: three trials raised some concerns, one trial was at high risk of bias, and two trial had low risk of bias. Most of the 'Risk of bias' judgements were at moderate to high risk of bias.

*Secondary outcomes:* Global function was measured using the Clinical Global Impression (CGI) instrument in five studies [16,26,27,30,31] and the Clinician Interview Based Impression of Change (CIBIC+) in five other studies [14,28,32,34,35]. Five studies were excluded from the meta-analysis: one study used an active control [34]; one study evaluated the effect of a different dose (20 ml) [14]; and 3 studies reported results that could not be incorporated [28,30,35]. The remaining RCTs evaluated the effect of 30 ml/day of Intravenous Cerebrolysin [16,26,27,31,32]. We dichotomized global performance into: ‘improved’, where participants demonstrated any improvement from baseline and ‘no improvement’ where participants demonstrated no change or worsened, following treatment. Across 479 participants from four studies, of which 248 received Cerebrolysin, there was improved global function in favor of Cerebrolysin OR 2.64 (95%CI:1.17 to 5.98; *P* = 0.02; 3C). There was substantial heterogeneity (*I^2^* = 74%). Funnel plot analysis was not feasible due to the limited number of included studies. The certainty of evidence was judged to be very low due to very serious RoB, inconsistency and imprecision (Table 2).

BPSD was assessed using a variety of outcome tools. The change in the BPSD was measured by numerous instruments, including the Sandoz Clinical Assessment-Geriatric (SCAG) scale in 3 studies [31,27,16]; the Neuropsychiatric Inventory (NPI) in 2 trials [32,34]; the Hamilton Depression scale (HAMD) in 2 studies [31,16]; the Montgomery Asberg Depression Rating Scale (MADR-S) in one study [30]; and Behavioral Symptoms in Alzheimer's Disease (Behave-AD) in one study [35]. One study used an active control [34]; one study reported no significant effects but did not provide data [30]; one study reported a significant difference between study groups in favor of Cerebrolysin but did not provide data [27]; and one study did not report the outcome assessment results [35]. Results from the 3 remaining studies with 427 participants, revealed a SMD of -0.11-point improvement in favor of Cerebrolysin (95%CI:-0.26 to 0.03; *P* = 0.13; Fig. 3D) with consistent results (*I*^2^ = 0). Funnel plot analysis was not feasible due to the limited number of included studies. The certainty of evidence was judged to be very low due to very serious RoB and imprecision (Table 2).

ADL was assessed using a variety of outcome tools. Studies were excluded from quantitative analysis for using an active control [34]; using a different dose (20 ml) [14]; reporting a significant difference between study groups in favor of Cerebrolysin without providing quantitative data [26,27]; and not reporting the ADL outcome data [35]. For the remaining 6 studies, with 809 participants, results indicated a SMD of -0.31-point improvement in favor of Cerebrolysin (95%CI:-0.43 to -0.19; *P* < 0.00001; Fig 3E). There was substantial heterogeneity (*I^2^* = 83%). The certainty of evidence was judged to be very low due to serious RoB, inconsistency, imprecision, and suspected publication bias (Table 2).

None of the included trials assessed quality of life or caregiver burden.

*Safety:* Seven studies, 1147 participants, reported sufficient detail to be included in meta-analyses of safety [14,30–32,34,35]. One study was excluded because an active control was used [34]. There was no apparent difference in incidence of SAE between study arms (Cerebrolysin:4.9% vs. control:4.0%; RR:1.16, 95%CI:0.67 to 2.02; *P* = 0.59; Fig. 3F) with consistent results (*I*^2^ = 0). Funnel plot analysis was not feasible due to the limited number of included studies. The certainty of evidence was judged to be low due to serious RoB and imprecision (Table 2).

**Fig. 4:**
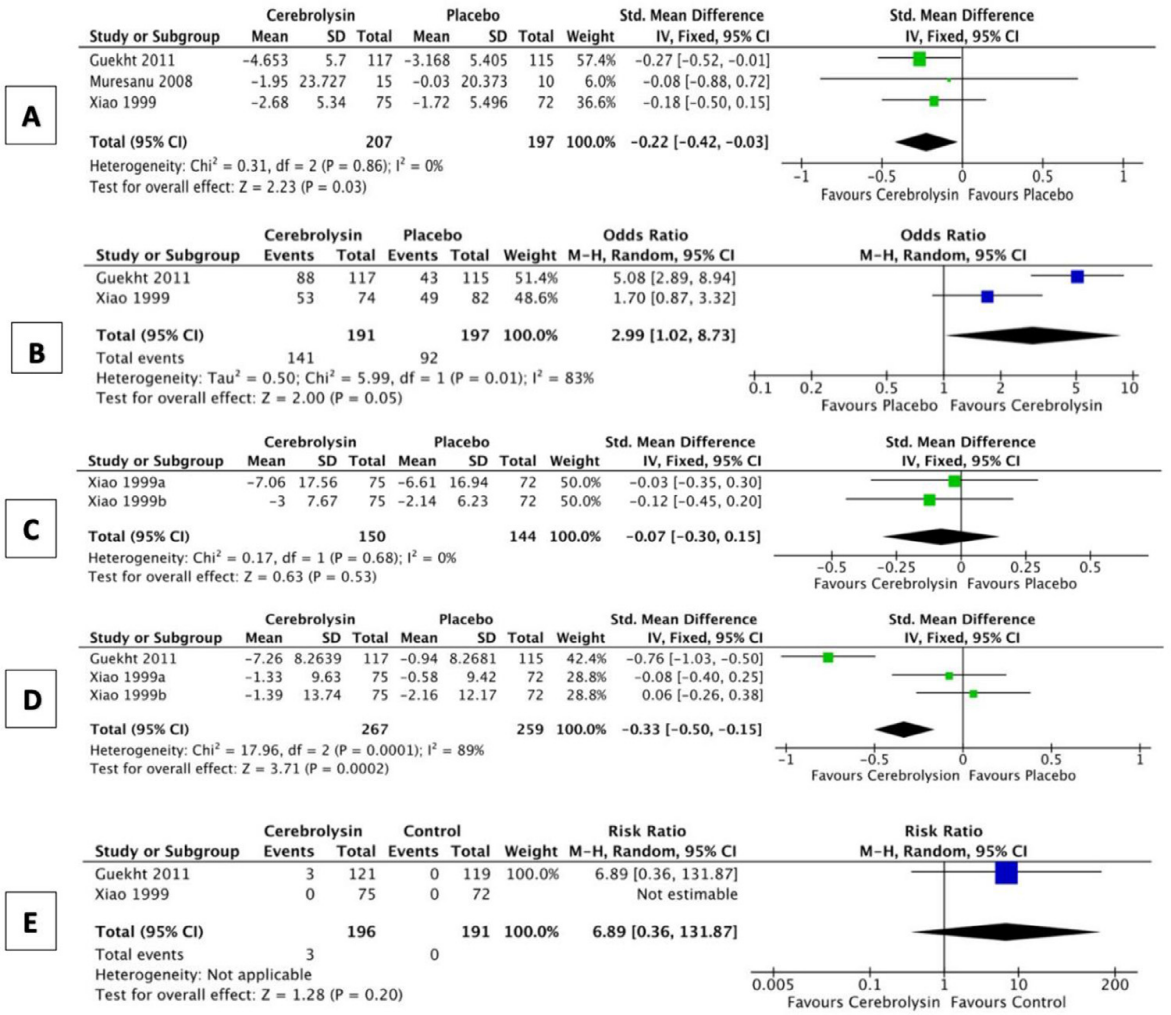
Forest plots comparing the change of various outcomes from baseline in patients with vascular cognitive impairment receiving Cerebrolysin vs. placebo, at the final follow-up visit. Cognition (A); global function(B); BPSD (C); ADL (D); the incidence of serious adverse events (E).

*Subgroups:* We performed subgroup analyses to assess Cerebrolysin in VCI. For cognitive function, there were 3 trials, with 404 participants, results revealed a SMD of -0.22 point improvement in favor of Cerebrolysin (95%CI:-0.42 to -0.03; *P* = 0.03; Fig. 4A). For clinical global impression, 2 trials, with 388 participants, there was improvement in favor of Cerebrolysin OR 2.99 (95%CI:1.02 to 8.73; *P* = 0.05; Fig. 4B). For BPSD, results from only 1 trial, with 147 participants, suggested no apparent effect, with a SMD of -0.07-point (95%CI:-0.30 to 0.15; *P* = 0.53; Fig. 4C). For ADL, there were 2 trials, with 379 participants, results suggested a SMD of -0.33 point improvement in favor of Cerebrolysin (95%CI:-0.50 to -0.15; *P* = 0.0003; Fig. 4D), however there was substantial imprecision in the estimate. There was apparent increased incidence of SAEs with Cerebrolysin, however there was substantial imprecision in the estimate with only 1 trial, with 240 participants (RR 6.89, 95%CI:0.36 to 131.87; *P* = 0.20; Fig. 4E). None of the included trials assessed quality of life or caregiver burden.

#### 3.3.2 Actovegin

*Primary outcome:* In one trial, with 503 participants, cognition assessed using ADAS-Cog+ demonstrated improvement in favor of Actovegin at 6 months (difference:-2.3, 95%CI:-3.9 to -0.7; *P* = 0.005) and 12 months (-3.7, 95%CI:-5.5 to -1.9; *P* < 0.001). Similarly, using Montreal Cognitive Assessment (MoCA) there was apparent improvement in favor of Actovegin at 6 months (0.7, 95%CI:0.2 to 1.3; *P* = 0.013) and 12 months (1.0, 95%CI:0.3 to 1.3; *P* = 0.003) [8].

*Secondary outcomes:* Change in clinical global function was assessed using the CGI instrument in one study, 60 participants. At four weeks, there was an apparent difference in favor of Cerebrolysin OR 7.4 (95%CI:1.89 to 28.62; *P* = 0.004) [33]. BPSD was assessed in one study using the SCAG, 58 participants [33]; and depressive symptoms in one study using the Beck Depression Inventory-II (BDI-II), 503 participants [8]. At four weeks, there was apparent improvement in BPSD in favor of Actovegin (difference:-10.8; *P* < 0.01) [33]. However, there was no apparent difference in depression at 6 and 12 months (Actovegin:60.9 and 62.1%; control:59.5%, and 55.3%, respectively) [8]. None of the included studies assessed ADL. QoL was assessed using the EQ-5D tool in one trial, 503 participants. The study reported no difference, at 6 and 12 months [8]. None of the included studies assessed the caregiver burden.

*Safety:* Incidence of SAEs was investigated in one study, 503 participants. There was no apparent difference but there was imprecision in the estimate (RR:2.17, 95% CI: 0.90 to 5.23; *P* = 0.8) [8].

#### 3.3.3 Cortexin

*Primary outcome:* The included study, 80 participants, used MMSE and MoCA scales. At twelve months, the number of patients with MoCA scores over 26 increased from baseline by 27.5% in favour of Cortexin. Results of MMSE assessment scores were not provided [13].

*Secondary outcomes:* Clinical global function, BPSD, ADL, QoL and caregiver burden were not assessed.

*Safety:* None of the treated participants reported any AEs or discontinuation [13].

## 4 Discussion

Our results suggest potential beneficial effects of animal derived nootropics, but evidence was not strong enough to allow for treatment recommendations or changes to guidelines. For Cerebrolysin there were significant improvements in cognitive and global function and the safety profile was reassuring. However, for many reasons these data are not definitive evidence of the efficacy of Cerebrolysin. The narrative results also suggest beneficial effects of Actovegin and Cortexin but the strength of evidence was even weaker than Cerebrolysin. Although the quantitative data are encouraging, most of the included papers had potential risk of bias issues, reducing the overall strength of evidence. The effects on efficacy outcomes were modest and would be regarded by many as smaller than the minimal important clinical difference. We also note the limited data on duration of benefit beyond the first months.

In general, the same pattern of potential benefit but no definitive data was seen when the analyses were restricted to people with VCI. There was a suggestion of possible greater size of cognitive effect of Cerebrolysin in the VCI populations but in contrast to the main analysis, in VCI there was a suggestion of higher incidence of SAEs with Cerebrolysin, albeit this was driven by one trial [14]. The Actovegin and Cortexin data were primarily based on stroke trials and so could be considered a VCI population. If further trials of nootropics are planned, a VCI focus would seem sensible.

Our findings align with other original research and systematic reviews on nootropics, albeit our review is broader in scope and includes contemporary studies. A previous review that considered Cerebrolysin specifically for Alzheimer's disease also found potential beneficial effects although again size of effect was modest [36]. Studies of Cerebrolysin in other neurological conditions are relevant and we note recent reviews reporting no clinical benefits in ischemic stroke and vascular dementia [37,38], but a suggestion of benefit in traumatic brain injury [39]. For Actovegin and Cortexin, there have been previous non-systematic, narrative reviews describing efficacy and safety, although results in stroke populations have been mixed [7,40,41]. It is notable that for both these nootropics there are more reviews than there are original research RCTs.

Our review offers an updated, comprehensive systematic search of available literature, using validated search filters and searching the international published data. We used new approaches to assessing risk of bias and frame our data using best practice approaches as recommended for guidelines. Despite our comprehensive search, the number of RCTs was limited and this precluded certain analytical approaches, for example funnel plots for publication bias, were not possible for all nootropics. However, as the certainty of evidence was judged to be low or very low before assessment for publication bias the lack of these analyses is unlikely to influence results.

Ultimately, our results do not support the recommendation of these drugs in healthcare systems where they are not used, but neither do our results definitively suggest that these drugs should be withdrawn in those settings where they are commonly used. Factors other than efficacy may need to be considered in the assessment of these drugs. As Cerebrolysin and Actovegin require frequent intravenous infusions there are issues the treatment burden, the economic and the opportunity cost of administration. With limited medium to longer term follow-up data, we do not know how frequently ‘courses’ of these drugs would need to be prescribed. However, given the lack of treatments for vascular and other neurocognitive disorders, some may argue that a modest benefit may be worth the cost.

To allow incorporation into guidelines further RCTs are needed. Our GRADE assessment of the published data can inform the design of any future trial. Contemporary trials would mandate a protocol and reporting according to best practice, these features were not seen in some of the historical trials included in this review. Future trials should use a standardized dosing regime and collect short, medium- and longer-term data. Outcomes should include health related quality of life and resource use, including need for care-home as these data would be needed for economic modelling. For consistency with other cognitive trials of an investigational medicinal product, caregiver derived outcomes should be considered. Finally, future trials should have a sample size large enough to detect a clinically meaningful difference in the outcomes of interest.

## 5 Conclusion

Despite the extensive use of animal-derived nootropics across international healthcare settings, the current published evidence regarding the efficacy and safety of these drugs in cognitive impairment, including VCI, is limited. Although studies assessing animal-derived nootropics suggested a favorable benefit-risk ratio, methodological limitations weaken the strength of evidence. To allow a definitive recommendation on these agents would require further RCTs using best practice in trial design and reporting, with larger sample sizes and longer follow-up periods.

## Appendix Supplementary materials

Image, application 1

## References

[1] World Health Organization (2017).

[2] Wimo A. (2017). The worldwide costs of dementia 2015 and comparisons with 2010. Alzheimers Dement..

[3] Sun M.K. (2018). Potential therapeutics for vascular cognitive impairment and dementia. Curr. Neuropharmacol..

[4] Colucci L. (2012). Effectiveness of nootropic drugs with cholinergic activity in treatment of cognitive deficit: a review. J. Exp. Pharmacol..

[5] Suliman N.A. (2016). Establishing Natural nootropics: recent molecular enhancement influenced by natural nootropic. Evid. Based Complement. Alternat. Med..

[6] Shabanov P.D. (2007). Comparison of behavioral effects of cortexin and cerebrolysin injected into brain ventricles. Bull. Exp. Biol. Med..

[7] Zavaliy L.B., Petrikov S.S., Schegolev A.V. (2018). Metabolic therapy in patients with ischemic stroke. Russ. Sklifosovsky J. Emerg. Med. Care.

[8] Guekht A. (2017). ARTEMIDA trial (A randomized trial of efficacy, 12 months international double-blind actovegin): a randomized controlled trial to assess the efficacy of actovegin in poststroke cognitive impairment. Stroke.

[9] Guekht A. (2013). A randomised, double-blind, placebo-controlled trial of actovegin in patients with post-stroke cognitive impairment: ARTEMIDA study design. Dement. Geriatr. Cogn. Dis. Extra.

[10] Yakovlev A.A. (2017). [Peptide drug cortexin inhibits brain caspase-8]. Biomed. Khim..

[11] Cui S. (2019). Cerebrolysin for vascular dementia. Cochrane Database Syst. Rev..

[12] Plosker G.L., Gauthier S. (2009). Cerebrolysin: a review of its use in dementia. Drugs Aging.

[13] Evzel'man M.A., Aleksandrova N.A. (2015). Cognitive impairments in patients with ischemic stroke and their correction. Neurosci. Behav. Physiol..

[14] Guekht A.B. (2011). Cerebrolysin in vascular dementia: improvement of clinical outcome in a randomized, double-blind, placebo-controlled multicenter trial. J. Stroke Cerebrovasc. Dis..

[15] Muresanu D.F. (2008). A pilot study to evaluate the effects of Cerebrolysin on cognition and qEEG in vascular dementia: cognitive improvement correlates with qEEG acceleration. J. Neurol. Sci..

[16] Xiao S., Yan H., Yao P. (1999). The efficacy of cerebrolysin in patients with vascular dementia: results of a Chinese multicentre, randomised, double-blind, placebo-controlled trial. Hong Kong J. Psychiatry.

[17] Liberati A. (2009). The PRISMA statement for reporting systematic reviews and meta-analyses of studies that evaluate healthcare interventions: explanation and elaboration. BMJ.

[18] Moher D. (2009). Preferred Reporting items for systematic reviews and meta-analyses: the PRISMA statement. PLOS Med..

[19] Shenkin S.D. (2017). Systematic reviews: guidance relevant for studies of older people. Age Ageing.

[20] Elsevier (2019).

[21] Sterne J.A.C. (2019). RoB 2: a revised tool for assessing risk of bias in randomised trials. BMJ.

[22] McGuinness L.A., Higgins J.P.T. (2020). Risk-of-bias VISualization (robvis): an R package and shiny web app for visualizing risk-of-bias assessments. Res. Synth. Methods.

[23] R.M.R.C. program], (2020) RevMan. The Cochrane Collaboration. Version 5.4. .

[24] R. Ryan and S. Hill. How to grade the quality of the evidence. 2016 3 September 2020]; Available from: http://cccrg.cochrane.org/author-resources.

[25] G. GDT. GRADEpro Guideline Development Tool. 2020; Available from: https://gradepro.org/.

[26] Bae C.Y. (2000). A double-blind, placebo-controlled, multicenter study of Cerebrolysin for Alzheimer's disease. J. Am. Geriatr. Soc..

[27] Rüther E. (1994). Efficacy of the peptidergic nootropic drug cerebrolysin in patients with senile dementia of the Alzheimer type (SDAT). Pharmacopsychiatry.

[28] Muresanu D.F., Rainer M., Moessler H. (2002). Improved global function and activities of daily living in patients with AD: a placebo-controlled clinical study with the neurotrophic agent Cerebrolysin. J. Neural Transm. Suppl..

[29] Chen C.C. (2013). Cerebrolysin enhances cognitive recovery of mild traumatic brain injury patients: double-blind, placebo-controlled, randomized study. Br. J. Neurosurg..

[30] Ruether E. (2001). A 28-week, double-blind, placebo-controlled study with Cerebrolysin in patients with mild to moderate Alzheimer's disease. Int. Clin. Psychopharmacol..

[31] Shifu X. (2000). Efficacy of FPF 1070 (cerebrolysin) in patients with Alzheimer's disease. A multicentre, randomised, double-blind, placebo-controlled trial. Clin. Drug Investig..

[32] Alvarez X.A. (2006). A 24-week, double-blind, placebo-controlled study of three dosages of cerebrolysin in patients with mild to moderate Alzheimer's disease. Eur. J. Neurol..

[33] Kanowski S. (1995). Confirmed clinical efficacy of actovegin in elderly patients with organic brain syndrome. Pharmacopsychiatry.

[34] Alvarez X.A. (2011). Combination treatment in Alzheimer's disease: results of a randomized, controlled trial with cerebrolysin and donepezil. Curr. Alzheimer Res..

[35] Panisset M. (2002). Cerebrolysin in Alzheimer's disease: a randomized, double-blind, placebo-controlled trial with a neurotrophic agent. J. Neural Transm..

[36] Wei Z.H. (2007). Meta-analysis: the efficacy of nootropic agent Cerebrolysin in the treatment of Alzheimer's disease. J. Neural Transm..

[37] Ziganshina L.E., Abakumova T., Vernay L. (2017). Cerebrolysin for acute ischaemic stroke. Cochrane Database Syst. Rev..

[38] Chen N. (2013). Cerebrolysin for vascular dementia. Cochrane Database Syst. Rev..

[39] Ghaffarpasand F. (2018). Effects of cerebrolysin on functional outcome of patients with traumatic brain injury: a systematic review and meta-analysis. Neuropsychiatr. Dis. Treat..

[40] Farooq M.U. (2017). Pharmacotherapy for vascular cognitive impairment. CNS Drugs.

[41] Skoog I., Korczyn A.D., Guekht A. (2012). Neuroprotection in vascular dementia: a future path. J. Neurol. Sci..

