# Supplementary material for: The efficacy and safety of animal-derived nootropics in cognitive disorders: Systematic review and meta-analysis
Source: Cereb Circ Cogn Behav. 2021 Apr 16;2:100012. doi: 10.1016/j.cccb.2021.100012 (PMC9616232; doi:10.1016/j.cccb.2021.100012)
Supplement: Supplementary file 1 [file mmc1.docx]

**Supplementary Materials:**

**Table I. Sources searched and details of the search strategies**

| Source | Search strategy |
| --- | --- |
| Database(s): Ovid MEDLINE(R) and In-Process & Other Non-Indexed Citations 1946 to August 24, 2020  [Date of most recent search: 25 August 2020] | 1. exp Dementia/  2. Delirium/  3. Wernicke Encephalopathy/  4. Delirium, Dementia, Amnestic, Cognitive Disorders/  5. dement*.mp.  6. alzheimer*.mp.  7. (lewy* adj2 bod*).mp.  8. deliri*.mp.  9. (chronic adj2 cerebrovascular).mp.  10. ("organic brain disease" or "organic brain syndrome").mp.  11. ("normal pressure hydrocephalus" and "shunt*").mp.  12. "benign senescent forgetfulness".mp.  13. (cerebr* adj2 deteriorat*).mp.  14. (cerebral* adj2 insufficient*).mp.  15. (pick* adj2 disease).mp.  16. (creutzfeldt or jcd or cjd).mp.  17. huntington*.mp.  18. binswanger*.mp.  19. korsako*.mp.  20. or/1‐19  21. Randomized Controlled Trials as Topic/  22. randomized controlled trial/  23. Random Allocation/  24. Double Blind Method/  25. Single Blind Method/  26. clinical trial/  27. clinical trial, phase [i.pt](http://i.pt)  28. clinical trial, phase [ii.pt](http://ii.pt)  29. clinical trial, phase [iii.pt](http://iii.pt)  30. clinical trial, phase [iv.pt](http://iv.pt)  31. controlled clinical [trial.pt](http://trial.pt)  32. randomized controlled [trial.pt](http://trial.pt)  33. multicenter [study.pt](http://study.pt)  34. clinical [trial.pt](http://trial.pt)  35. exp Clinical Trials as topic/  36. or/21-35  37. (clinical adj trial$).tw  38. ((singl$ or doubl$ or treb$ or tripl$) adj (blind$3 or mask$3)).tw  39. PLACEBOS/  40. placebo$.tw  41. randomly [allocated.tw](http://allocated.tw)  42. (allocated adj2 random$).tw  43. or/37-42  44. 36 or 43  45. case [report.tw](http://report.tw)  46. letter/  47. historical article/  48. or/45-47  49. 44 not 48  50. 20 and 49  51. Cerebrolysin*.mp.  52. CERE.mp.  53. "FPF 1070”.mp.  54. or/51-53  55. 50 and 54 |

**Table II. Sources searched and details of the search strategies**

| Source | Search strategy |
| --- | --- |
| Database(s): Ovid MEDLINE(R) and In-Process & Other Non-Indexed Citations 1946 to August 24, 2020  [Date of most recent search: 25 August 2020] | 1. exp Dementia/  2. Delirium/  3. Wernicke Encephalopathy/  4. Delirium, Dementia, Amnestic, Cognitive Disorders/  5. dement*.mp.  6. alzheimer*.mp.  7. (lewy* adj2 bod*).mp.  8. deliri*.mp.  9. (chronic adj2 cerebrovascular).mp.  10. ("organic brain disease" or "organic brain syndrome").mp.  11. ("normal pressure hydrocephalus" and "shunt*").mp.  12. "benign senescent forgetfulness".mp.  13. (cerebr* adj2 deteriorat*).mp.  14. (cerebral* adj2 insufficient*).mp.  15. (pick* adj2 disease).mp.  16. (creutzfeldt or jcd or cjd).mp.  17. huntington*.mp.  18. binswanger*.mp.  19. korsako*.mp.  20. or/1‐19  21. Randomized Controlled Trials as Topic/  22. randomized controlled trial/  23. Random Allocation/  24. Double Blind Method/  25. Single Blind Method/  26. clinical trial/  27. clinical trial, phase [i.pt](http://i.pt)  28. clinical trial, phase [ii.pt](http://ii.pt)  29. clinical trial, phase [iii.pt](http://iii.pt)  30. clinical trial, phase [iv.pt](http://iv.pt)  31. controlled clinical [trial.pt](http://trial.pt)  32. randomized controlled [trial.pt](http://trial.pt)  33. multicenter [study.pt](http://study.pt)  34. clinical [trial.pt](http://trial.pt)  35. exp Clinical Trials as topic/  36. or/21-35  37. (clinical adj trial$).tw  38. ((singl$ or doubl$ or treb$ or tripl$) adj (blind$3 or mask$3)).tw  39. PLACEBOS/  40. placebo$.tw  41. randomly [allocated.tw](http://allocated.tw)  42. (allocated adj2 random$).tw  43. or/37-42  44. 36 or 43  45. case [report.tw](http://report.tw)  46. letter/  47. historical article/  48. or/45-47  49. 44 not 48  50. 20 and 49  51. Actovegin.mp.  52. Solcoseryl.mp.  53. highly purified calf hemodialysate.mp.  54. or/51-53  55. 50 and 54 |

**Table III. Sources searched and details of the search strategies**

| Source | Search strategy |
| --- | --- |
| Database(s): Ovid MEDLINE(R) and In-Process & Other Non-Indexed Citations 1946 to August 24, 2020  [Date of most recent search: 25 August 2020] | 1. exp Dementia/ 2. Delirium/ 3. Wernicke Encephalopathy/ 4. Delirium, Dementia, Amnestic, Cognitive Disorders/ 5. dement*.mp. 6. alzheimer*.mp. 7. (lewy* adj2 bod*).mp. 8. deliri*.mp. 9. (chronic adj2 cerebrovascular).mp. 10. ("organic brain disease" or "organic brain syndrome").mp. 11. ("normal pressure hydrocephalus" and "shunt*").mp. 12. "benign senescent forgetfulness".mp. 13. (cerebr* adj2 deteriorat*).mp. 14. (cerebral* adj2 insufficient*).mp. 15. (pick* adj2 disease).mp. 16. (creutzfeldt or jcd or cjd).mp. 17. huntington*.mp. 18. binswanger*.mp. 19. korsako*.mp. 20. or/1‐19 21. Randomized Controlled Trials as Topic/ 22. randomized controlled trial/ 23. Random Allocation/ 24. Double Blind Method/ 25. Single Blind Method/ 26. clinical trial/ 27. clinical trial, phase [i.pt](http://i.pt) 28. clinical trial, phase [ii.pt](http://ii.pt) 29. clinical trial, phase [iii.pt](http://iii.pt) 30. clinical trial, phase [iv.pt](http://iv.pt) 31. controlled clinical [trial.pt](http://trial.pt) 32. randomized controlled [trial.pt](http://trial.pt) 33. multicenter [study.pt](http://study.pt) 34. clinical [trial.pt](http://trial.pt) 35. exp Clinical Trials as topic/ 36. or/21-35 37. (clinical adj trial$).tw 38. ((singl$ or doubl$ or treb$ or tripl$) adj (blind$3 or mask$3)).tw 39. PLACEBOS/ 40. placebo$.tw 41. randomly [allocated.tw](http://allocated.tw) 42. (allocated adj2 random$).tw 43. or/37-42 44. 36 or 43 45. case [report.tw](http://report.tw) 46. letter/ 47. historical article/ 48. or/45-47 49. 44 not 48 50. 20 and 49 51. Cortexin*.mp. 52. 50 and 51 |


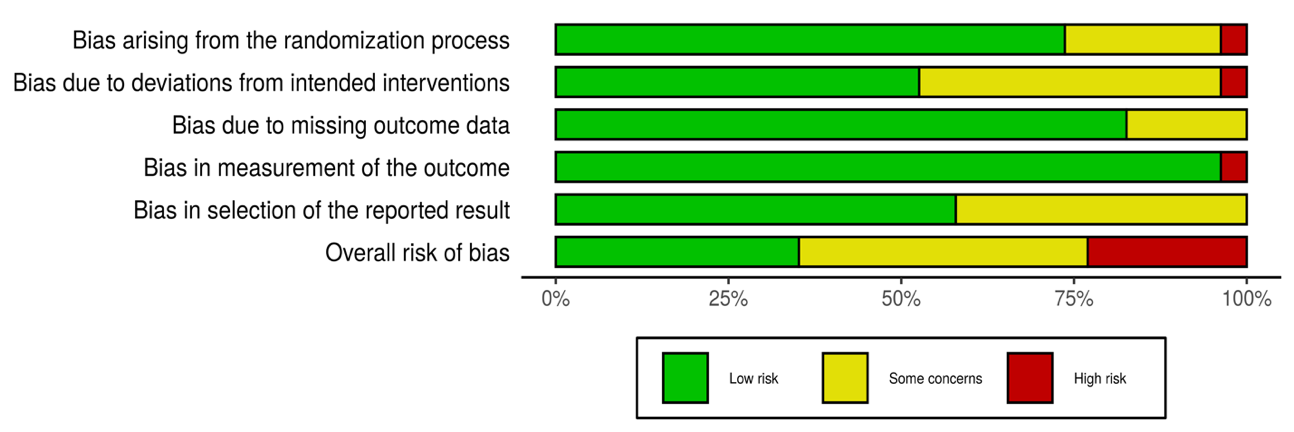


**Figure I.** Summary of risk of bias ratings presented as percentages of each domain’s contribution to the overall risk of bias across all included Cerebrolysin trials.

**
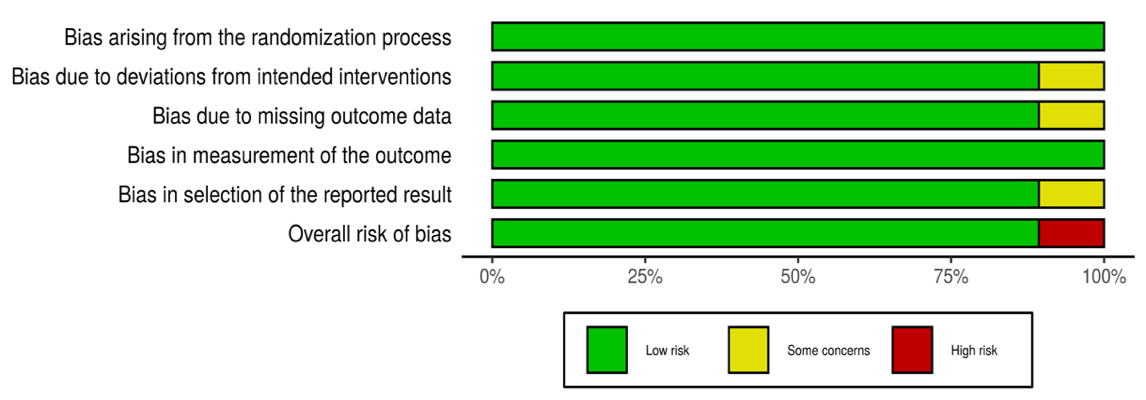
**

**Figure II.** Summary of risk of bias ratings presented as percentages of each domain’s contribution to the overall risk of bias across all included Actovegin trials.

**
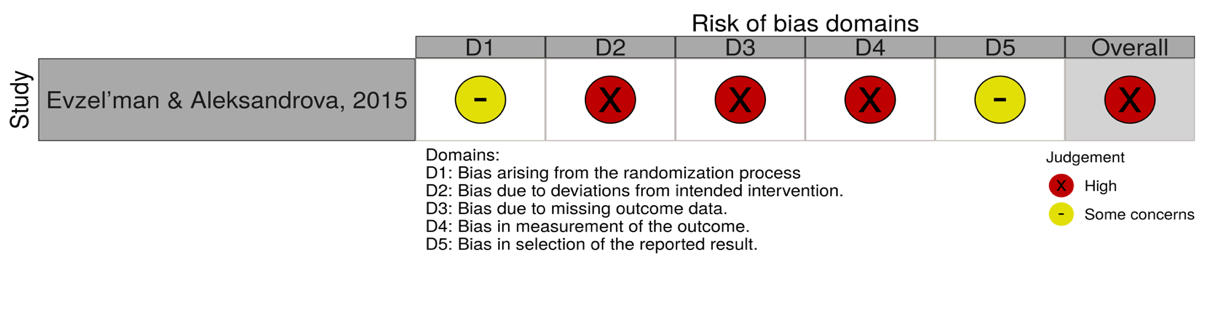
**

**Figure III.** Summary of risk of bias ratings of each bias domain for the included Cortexin trial.


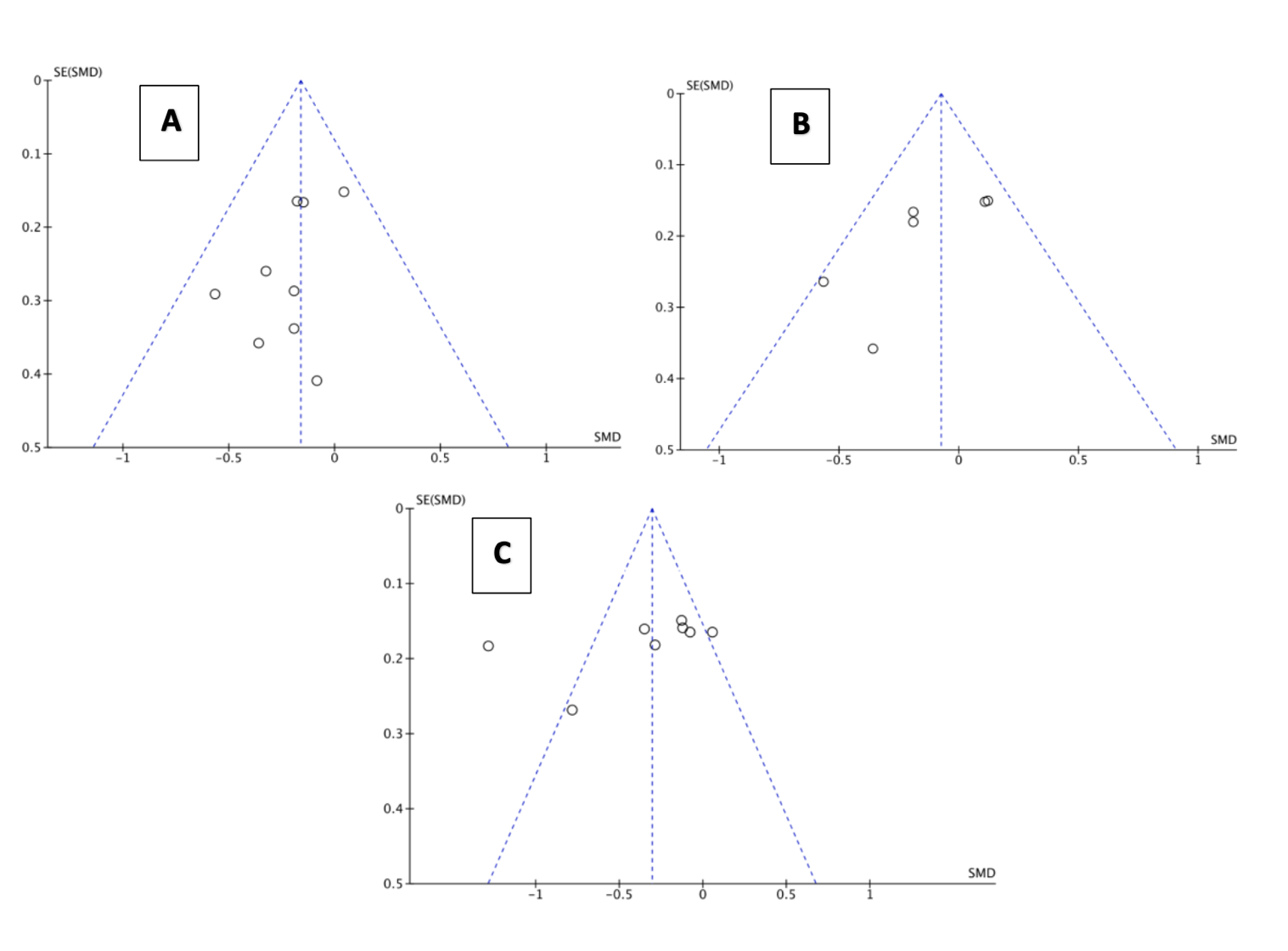


**Figure VI.** Funnel plots of Cerebrolysin vs. placebo for the following outcomes: (A) The change in cognitive function from baseline 3 to 4 weeks following the initiation of therapy; (B) The change in cognitive function from baseline 3 to 7 months following the initiation of therapy; (C) The change in activities of daily living from baseline at the last follow-up visit. Substantial asymmetry is observed in all funnel plots, indicating the potential risk of publication bias.
